# Supplementary material for: The experience of European hospital-based health care workers on following infection prevention and control procedures and their wellbeing during the first wave of the COVID-19 pandemic
Source: PLoS One. 2022 Feb 7;17(2):e0245182. doi: 10.1371/journal.pone.0245182 (PMC8820620; doi:10.1371/journal.pone.0245182)
Supplement: S1 Fig — (DOCX) [file pone.0245182.s001.docx]

*This is supplementary material to the manuscript: “The experience of European hospital-based health care workers on following infection prevention and control procedures and their wellbeing during the first wave of the COVID-19 pandemic.”*

*Denise van Hout*, Paul Hutchinson, Marta Wanat, Caitlin Pilbeam, Herman Goossens, Sibyl Anthierens, Sarah Tonkin-Crine, Nina Gobat*

**E-mail corresponding author:* [*denise.van.hout@rivm.nl*](mailto:denise.van.hout@rivm.nl)

**S1 Fig.** Self-reported availability of personal protective equipment during most recent clinical shift.

**
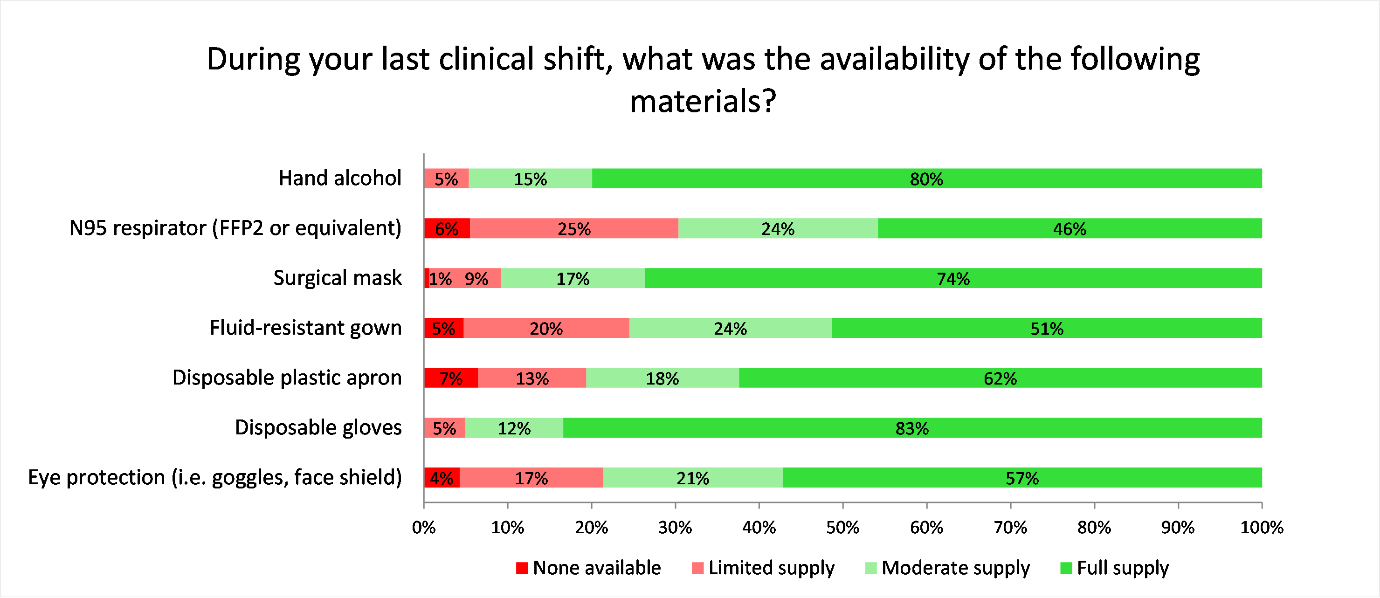
**

S1 Fig underlying data:

|  | N completed | None available | Limited supply | Moderate supply | Full supply |
| --- | --- | --- | --- | --- | --- |
| Eye protection (ie. goggles, face mask) | 2063 | 88 | 353 | 443 | 1179 |
| Disposable gloves | 2151 | 9 | 105 | 251 | 1786 |
| Disposable apron | 1875 | 122 | 241 | 342 | 1170 |
| Fluid-resistant gown | 2046 | 97 | 405 | 494 | 1050 |
| Surgical mask | 2156 | 14 | 184 | 370 | 1588 |
| N95 respirator (FFP2 or equivalent) | 2035 | 112 | 506 | 484 | 933 |
| Hand alcohol | 2168 | 9 | 116 | 317 | 1726 |
