# Supplementary material for: The experience of European hospital-based health care workers on following infection prevention and control procedures and their wellbeing during the first wave of the COVID-19 pandemic
Source: PLoS One. 2022 Feb 7;17(2):e0245182. doi: 10.1371/journal.pone.0245182 (PMC8820620; doi:10.1371/journal.pone.0245182)
Supplement: S1 Table — (DOCX) [file pone.0245182.s002.docx]

*This is supplementary material to the manuscript: “The experience of European hospital-based health care workers on following infection prevention and control procedures and their wellbeing during the first wave of the COVID-19 pandemic.”*

*Denise van Hout*, Paul Hutchinson, Marta Wanat, Caitlin Pilbeam, Herman Goossens, Sibyl Anthierens, Sarah Tonkin-Crine, Nina Gobat*

**E-mail corresponding author:* [*denise.van.hout@rivm.nl*](mailto:denise.van.hout@rivm.nl)

| **S1 Table.** Country of work of responding hospital healthcare workers (HCWs). | | |
| --- | --- | --- |
|  | **Round 1**  **N = 190 (%)** | **Round 2**  **N = 2099 (%)** |
| Albania | - | 7 (0.3) |
| Austria | 1 (0.5) | 3 (0.1) |
| Belarus | - | 2 (0.1) |
| Belgium | 4 (2.1) | 43 (2.0) |
| Bosnia and Herzegovina | - | 2 (0.1) |
| Bulgaria | 2 (1.2) | 3 (0.1) |
| Croatia | 8 (4.2) | 2 (0.1) |
| Cyprus | 1 (0.5) | 9 (0.4) |
| Czech Republic | 1 (0.5) | 3 (0.1) |
| Denmark | 3 (1.6) | 4 (0.2) |
| Estonia | 3 (1.6) | 29 (1,4) |
| Finland | - | 1 (0.1) |
| France | 8 (4.2) | 166 (7.9) |
| Germany | 6 (3.2) | 159 (7.6) |
| Greece | 23 (12) | 42 (2.0) |
| Hungary | 6 (3.2) | 7 (0.3) |
| Ireland | 2 (1.1) | 5 (0.2) |
| Israel | - | 4 (0.2) |
| Italy | 7 (3.7) | 54 (2.6) |
| Latvia | 3 (1.6) | 24 (1.1) |
| Lithuania | 1 (0.5) | - |
| Luxembourg | - | 1 (0.1) |
| Macedonia | 3 (1.6) | 3 (0.1) |
| Malta | - | 147 (7.0) |
| Montenegro | 1 (0.5) | - |
| Netherlands | 13 (6.8) | 242 (11.5) |
| Norway | - | 183 (8.7) |
| Poland |  | 13 (0.6) |
| Portugal | 18 (9.5) | 712 (33.9) |
| Republic of Kosovo | - | 2 (0.1) |
| Romania | 9 (4.7) | 8 (0.4) |
| Serbia | 1 (0.5) | 33 (1.6) |
| Slovakia | - | 1 (0.1) |
| Slovenia | 1 (0.5) | 1 (0.1) |
| Spain | 55 (28.9) | 98 (4.7) |
| Sweden | - | 2 (0.1) |
| Switzerland | - | 12 (0.6) |
| Turkey | 1 (0.5) | 9 (0.4) |
| Ukraine | - | 2 (0.1) |
| United Kingdom | 8 (4.2) | 61 (2.9) |
| Unknown | 1 (0.5) | - |
