# Supplementary material for: The experience of European hospital-based health care workers on following infection prevention and control procedures and their wellbeing during the first wave of the COVID-19 pandemic
Source: PLoS One. 2022 Feb 7;17(2):e0245182. doi: 10.1371/journal.pone.0245182 (PMC8820620; doi:10.1371/journal.pone.0245182)
Supplement: S2 Table — (DOCX) [file pone.0245182.s003.docx]

| **S2 Table.** Round 1 survey: comparison overall invited group of health care workers and those who responded. | | | |
| --- | --- | --- | --- |
|  | *Recipients survey*  *N = 2436 (%)* | *Respondents*  *N = 190 (%)* | *P value*^1^ |
| **Female** | 921 (37.8) | 84 (42.6) | NS |
| **Region^2^**  Eastern Europe  Northern Europe  Southern Europe  Western Europe  Unknown | 384 (15.8)  248 (10.2)  1083 (44.5)  715 (29.4)  6 (0.2) | 18 (9.5)  20 (10.5)  119 (62.6)  32 (16.8)  1 (0.5) | <.05 |
| **Medical specialty**  Acute care (anaesthesiology, ER, ICU)  Internal medicine  *Of which infectious diseases*  Public health & research  Surgery  Other  Unknown | 1068 (43.8)  885 (36.3)  *586 (24.1)*  196 (8.0)  181 (7.4)  69 (2.8)  37 (1.5) | 69 (36.3)  76 (40.0)  *36 (18.9)*  13 (6.8)  14 (7.4)  11 (5.8)  7 (3.7) | <.05 |
| ER, emergency room; ICU, intensive care unit; NS, non-significant ^1^ *C*omparison characteristics of responding sample to expected proportions based on overall survey recipient group, *P* value from Chi-square goodness of fit test. ^2^ Sub division of Europe adapted from the United Nations; for the current study, Cyprus, Israel and Turkey were categorized as Southern Europe (United Nations Statistics Division – Standard Country and Area Codes Classifications, accessible via <https://unstats.un.org/unsd/methodology/m49>). | | | |

*This is supplementary material to the manuscript: “The experience of European hospital-based health care workers on following infection prevention and control procedures and their wellbeing during the first wave of the COVID-19 pandemic.”*

*Denise van Hout*, Paul Hutchinson, Marta Wanat, Caitlin Pilbeam, Herman Goossens, Sibyl Anthierens, Sarah Tonkin-Crine, Nina Gobat*

**E-mail corresponding author:* [*denise.van.hout@rivm.nl*](mailto:denise.van.hout@rivm.nl)
