# Supplementary material for: The experience of European hospital-based health care workers on following infection prevention and control procedures and their wellbeing during the first wave of the COVID-19 pandemic
Source: PLoS One. 2022 Feb 7;17(2):e0245182. doi: 10.1371/journal.pone.0245182 (PMC8820620; doi:10.1371/journal.pone.0245182)
Supplement: S3 Table — (DOCX) [file pone.0245182.s004.docx]

*This is supplementary material to the manuscript: “The experience of European hospital-based health care workers on following infection prevention and control procedures and their wellbeing during the first wave of the COVID-19 pandemic.”*

*Denise van Hout*, Paul Hutchinson, Marta Wanat, Caitlin Pilbeam, Herman Goossens, Sibyl Anthierens, Sarah Tonkin-Crine, Nina Gobat*

**E-mail corresponding author:* [*denise.van.hout@rivm.nl*](mailto:denise.van.hout@rivm.nl)

| **S3 Table.** Multivariable logistic regression for the association between gender and a WHO-5 Well-being Index below 50 points, in hospital healthcare workers during the COVID-19 pandemic. | | | | |
| --- | --- | --- | --- | --- |
|  | **aOR** | **95% CI** | | ***P value*** |
| Age (in years) | 1.0 | 1.0 | 1.0 | .03 |
| Female gender | 1.5 | 1.2 | 1.8 | <.001 |
| Living alone | 1.2 | 0.9 | 1.6 | NS |
| Job role  Other  Junior nurse  Senior nurse  Junior medical doctor  Senior medical doctor  Junior allied health professional  Senior allied health professional | ref  1.0  1.0  1.0  1.1  1.0  1.5 | ref  0.7  0.7  0.6  0.7  0.5  0.9 | ref  1.6  1.5  1.5  1.5  2.3  2.8 | -  NS  NS  NS  NS  NS  NS |
| Academic hospital | 0.9 | 0.8 | 1.1 | NS |
| Region^1^  Western Europe  Eastern Europe  Southern Europe  Northern Europe | ref  3.6  2.3  1.8 | ref  2.0  1.8  1.3 | ref  6.6  2.9  2.4 | -  <.001  <.001  <.001 |
| Providing direct COVID-19 patient care | 1.3 | 1.0 | 1.6 | .04 |
| CI, confidence interval; COVID-19, coronavirus disease 2019; HCW, healthcare worker; OR, odds ratio; WHO, World Health Organization  ^1^ Sub division of Europe adapted from the United Nations; for the current study, Cyprus, Israel and Turkey were categorized as Southern Europe (United Nations Statistics Division – Standard Country and Area Codes Classifications, accessible via <https://unstats.un.org/unsd/methodology/m49>). | | | | |
