# Supplementary material for: The experience of European hospital-based health care workers on following infection prevention and control procedures and their wellbeing during the first wave of the COVID-19 pandemic
Source: PLoS One. 2022 Feb 7;17(2):e0245182. doi: 10.1371/journal.pone.0245182 (PMC8820620; doi:10.1371/journal.pone.0245182)
Supplement: S2 File — (DOCX) [file pone.0245182.s006.docx]

**Supporting information 2. Principal component analysis**

*This is supplementary material to the manuscript: “The experience of European hospital-based health care workers on following infection prevention and control procedures and their wellbeing during the first wave of the COVID-19 pandemic.”*

*Denise van Hout*, Paul Hutchinson, Marta Wanat, Caitlin Pilbeam, Herman Goossens, Sibyl Anthierens, Sarah Tonkin-Crine, Nina Gobat*

**E-mail corresponding author:* [*denise.van.hout@rivm.nl*](mailto:denise.van.hout@rivm.nl)

**Background**

We used principal component analysis of data collected in the first survey round to reduce the dimensionality of three important constructs into individual scales. The three scales correspond to measures of beliefs, skills, and availability of PPE. Prior to construction of the indices, we calculated Cronbach’s alpha to measure the internal consistency of the variables for each index. In our analysis, the Cronbach’s alphas ranged from 0.6958 to 0.8468. A Cronbach’s alpha of 0.70 is generally considered to be acceptable. In the one case where the alpha fell below that threshold, it was very close to the acceptable level.

For the indices, we used the first principal component since that is the component that maximizes the variance, yielding the largest eigenvalue of the covariance matrix. The proportion of the variance explained by the first principal component ranged from 0.4031 to 0.7718. Scoring coefficients were then used to construct index values for each observation for each index.

**Codebook**

| Variable | Survey statement |
| --- | --- |
| beliefs_capab | I am confident that I am able to follow recommended procedures related to personal protective equipment(PPE) for COVID-19 e.g. appropriate use and disposal of gloves, apron and fluid resistant surgical mask. |
| beliefs_eff | I consider that the implementation of protective procedures at work are effective to prevent the spread of COVID-19 in my hospital. |
| beliefs_protect | Following the infection prevention and control recommendations will protect me from becoming ill with COVID-19. |
| beliefs_sign_strain | Following recommended infection prevention and control procedures adds significant additional strain to my workload. |
| skills_general | I have received general training for infection, prevention and control procedures for communicable diseases |
| skills_covid19 | I have received **sufficient** training in the infection prevention and control practices specifically for COVID-19 |
| skills_confidence_ppe | I am confident in my ability to correctly don and doff personal protective equipment to prevent transmission of COVID-19 to others and myself. |
|  | During your last clinical shift, what was the availability of the following materials (none available, limited supply, moderate supply, full supply) |
| environm_av_hand | Hand alcohol |
| environm_av_n95 | N95 respirator (FFP2 or equivalent) |
| environm_av_surgical | Surgical mask |
| environm_av_apron | Disposable apron |
| environm_av_gloves | Disposable gloves |
| environm_av_suit | Full body suit |
| environm_av_eye | Eye protection (e.g., goggles or face shield) |

**Script PCA**

. alpha beliefs_capab beliefs_eff beliefs_protect beliefs_sign_strain

Test scale = mean(unstandardized items)

Average interitem covariance: .7189784

Number of items in the scale: 4

Scale reliability coefficient: 0.6958

. factor beliefs_capab beliefs_eff beliefs_protect beliefs_sign_strain, factors(1) pcf

(obs=184)

Factor analysis/correlation Number of obs = 184

Method: principal-component factors Retained factors = 1

Rotation: (unrotated) Number of params = 4

--------------------------------------------------------------------------

Factor | Eigenvalue Difference Proportion Cumulative

-------------+------------------------------------------------------------

Factor1 | 2.16156 1.21857 0.5404 0.5404

Factor2 | 0.94300 0.37481 0.2357 0.7761

Factor3 | 0.56818 0.24093 0.1420 0.9182

Factor4 | 0.32726 . 0.0818 1.0000

--------------------------------------------------------------------------

LR test: independent vs. saturated: chi2(6) = 176.41 Prob>chi2 = 0.0000

Factor loadings (pattern matrix) and unique variances

---------------------------------------

Variable | Factor1 | Uniqueness

-------------+----------+--------------

beliefs_ca~b | 0.7466 | 0.4427

beliefs_eff | 0.8736 | 0.2368

beliefs_pr~t | 0.8494 | 0.2785

beliefs_si~n | 0.3458 | 0.8805

---------------------------------------

. predict beliefs

(option regression assumed; regression scoring)

Scoring coefficients (method = regression)

------------------------

Variable | Factor1

-------------+----------

beliefs_ca~b | 0.34538

beliefs_eff | 0.40416

beliefs_pr~t | 0.39295

beliefs_si~n | 0.15996

------------------------

. alpha skills_general skills_covid19 skills_confidence_ppe

Test scale = mean(unstandardized items)

Average interitem covariance: 1.666164

Number of items in the scale: 3

Scale reliability coefficient: 0.8468

. factor skills_general skills_covid19 skills_confidence_ppe, factors(1) pcf

(obs=188)

Factor analysis/correlation Number of obs = 188

Method: principal-component factors Retained factors = 1

Rotation: (unrotated) Number of params = 3

--------------------------------------------------------------------------

Factor | Eigenvalue Difference Proportion Cumulative

-------------+------------------------------------------------------------

Factor1 | 2.31548 1.85154 0.7718 0.7718

Factor2 | 0.46394 0.24335 0.1546 0.9265

Factor3 | 0.22058 . 0.0735 1.0000

--------------------------------------------------------------------------

LR test: independent vs. saturated: chi2(3) = 268.06 Prob>chi2 = 0.0000

Factor loadings (pattern matrix) and unique variances

---------------------------------------

Variable | Factor1 | Uniqueness

-------------+----------+--------------

skills_gen~l | 0.8747 | 0.2349

skills_co~19 | 0.9252 | 0.1441

skills_con~e | 0.8333 | 0.3055

---------------------------------------

. predict skills

(option regression assumed; regression scoring)

Scoring coefficients (method = regression)

------------------------

Variable | Factor1

-------------+----------

skills_gen~l | 0.37776

skills_co~19 | 0.39955

skills_con~e | 0.35990

------------------------

. alpha environm_av_hand environm_av_n95 environm_av_surgical environm_av_apron environm_av_gloves environm_av_suit environm_av_eye

Test scale = mean(unstandardized items)

Average interitem covariance: .2197659

Number of items in the scale: 7

Scale reliability coefficient: 0.7115

. factor environm_av_hand environm_av_n95 environm_av_surgical environm_av_apron environm_av_gloves environm_av_suit environm_av_eye, factors(1) pcf

(obs=183)

Factor analysis/correlation Number of obs = 183

Method: principal-component factors Retained factors = 1

Rotation: (unrotated) Number of params = 7

--------------------------------------------------------------------------

Factor | Eigenvalue Difference Proportion Cumulative

-------------+------------------------------------------------------------

Factor1 | 2.82162 1.83552 0.4031 0.4031

Factor2 | 0.98610 0.23222 0.1409 0.5440

Factor3 | 0.75388 0.04749 0.1077 0.6517

Factor4 | 0.70640 0.02346 0.1009 0.7526

Factor5 | 0.68294 0.10816 0.0976 0.8501

Factor6 | 0.57478 0.10050 0.0821 0.9322

Factor7 | 0.47428 . 0.0678 1.0000

--------------------------------------------------------------------------

LR test: independent vs. saturated: chi2(21) = 231.60 Prob>chi2 = 0.0000

Factor loadings (pattern matrix) and unique variances

---------------------------------------

Variable | Factor1 | Uniqueness

-------------+----------+--------------

environm_a~d | 0.5774 | 0.6666

environm_~95 | 0.6852 | 0.5305

environm_a~l | 0.7352 | 0.4595

environm~ron | 0.6324 | 0.6000

environm_~es | 0.6083 | 0.6300

environm_a~t | 0.5227 | 0.7268

environm_a~e | 0.6595 | 0.5650

---------------------------------------

. predict environment

(option regression assumed; regression scoring)

Scoring coefficients (method = regression)

------------------------

Variable | Factor1

-------------+----------

environm_a~d | 0.20464

environm_~95 | 0.24285

environm_a~l | 0.26055

environm~ron | 0.22413

environm_~es | 0.21558

environm_a~t | 0.18526

environm_a~e | 0.23374

------------------------
