# Supplementary material for: The experience of European hospital-based health care workers on following infection prevention and control procedures and their wellbeing during the first wave of the COVID-19 pandemic
Source: PLoS One. 2022 Feb 7;17(2):e0245182. doi: 10.1371/journal.pone.0245182 (PMC8820620; doi:10.1371/journal.pone.0245182)
Supplement: Attachment — Submitted filename: 20211017 Response to reviewers.docx [file pone.0245182.s007.docx]

Reviewer #1: The study benefitted from a good sample size and from addressing topical and relevant issues (e.g., healthcare staff wellbeing and infection and prevention controls). However, I felt confused throughout as to what the main goals of the study were – it felt like a mishmash of questions had been asked and statistics had been conducted. I also felt concerned as to the factor analysis which is briefly referenced and minimally described but which is of key importance to the overall study (as it was the basis for developing the main questionnaire used in the study). In general, it seemed a lot of survey questions had been asked but not enough thought had been put into making the data into a clear and cohesive study. Specific comments below:

*Author’s response: We would sincerely like to thank the reviewer for their valuable time and we sincerely appreciate the careful reading of our manuscript. In the following we will address all comments point by point.*

Abstract:

1. The aims are not clear- what were the specific purposes of doing this survey? What did the authors want to learn? Were there any predictions?

*Author’s response*: *The study took place at a crucial time in the peak of the first wave of the pandemic in order to rapidly respond on an international level to the needs of the healthcare workers.* *The primary aim of the study was to capture health worker perceptions related to key factors that are known to influence their adherence to infection prevention and control guidelines. The study was rapidly developed at the start of the pandemic, and, in response to emerging evidence of the impact of the pandemic on health worker mental health and emotional well-being, a second objective was included to capture data related to wellbeing.*

*Changes to the manuscript: To clarify these objectives in the abstract, we have updated the text – please see p. 2, lines 29-32 of the revised manuscript.*

2. The reason for having two ‘rounds’ is also unclear. As all the results seem to only refer to one group, were the two ‘rounds’ simply two different ways of recruiting participants and the two groups were then pooled for the purposes of the analysis?

*Authors’ response: This was a rapidly developed study, designed to respond quickly to emerging needs at the start of the pandemic. The first round was conducted during the first peak pandemic wave in Europe. We had a low response rate to this survey at round 1 – in part due to competing clinical pressures during this time. For the second round of data collection, we adapted our approach to enhance recruitment.. This second round of data collection was undertaken toward the end of the first pandemic wave in Europe, and we had a strong response. Importantly, the target population of both rounds were the same, only our recruitment approach changed.*

*Changes to the manuscript: To clarify this further, we have updated the abstract – please see p. 2, lines 34-38 and we have added information to the methods section – please see p. 6, lines 117-121.*

Intro

The intro covers some relevant issues – for example, demonstrating that HCWs are at heightened risk of both catching Covid and experiencing burnout. It stresses that HCWs should be familiar with IPC procedures, and that women may be at higher risk of experiencing stress and burnout. However, there are a couple of issues with the introduction:

3. It is unclear what the gaps in knowledge are as the introduction progresses. The authors need to more clearly demonstrate what IS NOT known which the present research aimed to address

*Authors’ response: We thank the reviewer for this comment. We have added more specific statements about the research gaps that our study aimed to fulfill in to the Introduction section of the paper.*

*Changes to the manuscript: please see p. 4, lines 72-74 and p. 5, lines 95-97 of the track changes version of the manuscript.*

4. The aims are poorly written. They are extremely broad and do not give the reader a sense of where the analyses and results will be heading. It would be acceptable to have broad aims but to follow these up with specific objectives.

*Authors’ response: We thank the reviewer for this suggestion. We acknowledge the aims of our study were indeed broad and mostly descriptive of nature. We also agree that our specific objective to look at subgroups, such as potential differences in perceptions of male and female HCWs, were not clearly described to readers in the Introduction section. We have therefore updated the Introduction of the manuscript accordingly.*

*Changes to the manuscript: see p. 5, lines 101-105 of the track changes version of the manuscript.*

5. The use of a survey methodology also needs to be justified – both of the current aims strike me as being better suited to a qualitative study. Again, the gap in the literature that this study (using a survey approach) addresses needs to be clear.

*Authors’ response: Thank you for this remark. The choice of survey methodology was driven, in part, by a pragmatic need to rapidly collect data that could be used to inform operational response. The dimensions included in the survey linked with key factors that are known to influence adherence to infection control guidelines and which can then guide specific actions to improve staff adherence to guidelines. We agree that a qualitative study related to these issues would have provided rich insights into many of the issues raised. Indeed, many of the other groups that implemented this survey in other parts of the world as part of the WHO R&D Blueprint ‘COVID-19 Social Science in Outbreaks’ (the study protocol and survey tool are freely available), included a free text open comments box in their surveys and elicited interesting findings. However, in our case, by using a survey design we were able to acquire a large sample size and international reach, which would have been far smaller if we would have used a qualitative study. It is also important to note that during our study period, at the beginning of the COVID-19 pandemic in Europe, there was already a high burden on healthcare professionals. A benefit of survey methods is that they require much lower commitment for participants already under strain.*

*Changes to the manuscript: we have added the rationale for a survey study design to our Discussion, see p. 19, lines 376-381 of the track changes version of the manuscript.*

Methods

6. The section on the survey tool is confusing. It says the tool was developed ‘to capture theoretically informed influences on HCWs’ motivation, opportunity and ability to follow general IPC precautions’. There needs to be info on the number of items, how many captured each of these three things (motivation, opportunity and ability), and some example questions. There also needs to be basic info about the psychometrics of the survey once it had been developed for use in round 2 of the research (i.e., following the first round of the research). It is not sufficient to say the authors didn’t have this information to begin with.

*Authors’ response: This study was rapidly conceived and developed at the start of the COVID-19 pandemic to respond to a need to better understand how prepared health workers were to adhere to infection prevention and control guidelines. At the point of protocol development, a well-developed and pre-validated tool to capture behavioral determinants of guideline adherence was not in existence. We therefore rapidly developed a tool based on expert opinion and guided by the Theoretical Domains Framework. Data from the first round of data collection in this study were used to explore the psychometric properties of the tool, not only for the second round of sampling but also for groups implementing the same survey in different parts of the world. Please note that as mentioned in the paper, a Research Template of this study has been developed, including a generic version of the protocol and the survey tool. These materials are freely available via the Research & Development (R&D) COVID-19 Blueprint of the World Health Organization (WHO) to be used in different clinical settings globally. Link:* [*https://www.who.int/publications/m/item/perceptions-of-healthcare-workers-regarding-local-infection-prevention-and-control-procedures-for-covid-19-research-protocol*](https://www.who.int/publications/m/item/perceptions-of-healthcare-workers-regarding-local-infection-prevention-and-control-procedures-for-covid-19-research-protocol)*.*

*Changes to the manuscript: no changes were made to the manuscript, the information above is already depicted in the original paper, please see p. 6-7, lines 128-141 and p. 7, lines 143-147.*

7. The section on the survey tool is also confusing because at the end of the paragraph the authors throw in that the purpose of the first round of the survey was to develop and refine the tool which is used in round 2. This should have been clear from the abstract and the introduction. If such a tool (which is the foundation of the research) didn’t exist prior to the survey being conducted, then one of the aims of the study was surely to develop such a measure? Aside from this only one other questionnaire is used, so it is important that this is clarified.

*Authors’ response: We apologize for any confusion. The purpose of the first round of the survey was not specifically “to develop” the tool for a second round of sampling within our study (ie. the second round with different sampling method was performed because of low response rate). However, when deciding on a second round of sampling we did use data from the first round of data collection to explore the psychometric properties of the tool and to refine the items for data collection, also for groups implementing the same survey in different parts of the world. See also the explanation above. We hope this clarifies.*

8. The statistical analyses section refers to objectives – but these objectives are not outlined anywhere earlier in the paper. These objectives need to be provided at the end of the introduction. The methods is not the place to introduce new objectives.

*Authors’ response: Thank you for this comment. The respective statistical analyses section refers to the objectives stated at the end of the Introduction section of the paper, which were previously there referred to as ‘aims’. We acknowledge this was not clear to readers.*

*Changes to the manuscript: we have updated the text and clarified the objectives – please see p. 5, lines 101-105 of the track changes version of the manuscript).*

9. ‘bivariate analyses for gender, job role, European region and providing COVID-19 care by

independent sample T-test or one-way ANOVA’: when were independent sample T-tests used and when were ANOVAS used? This needs to be clarified

*Authors’ response*: *an independent sample T-test is carried out to investigate two groups, whereas ANOVA is used to compare multiple (>2) groups (ANOVA is equivalent to running multiple T-tests). We have clarified this in our manuscript.*

*Changes to the manuscript: please see p. 8, lines 166-167 of the track changes version of the manuscript).*

10. The statistical analyses section also describes conducting tests relating to examining gender etc differences in wellbeing and factors relating to ‘HCWs’ perceived sense of control over getting infected with COVID-19’. Due to unclear aims and objectives earlier in the paper, these analyses seem to ‘come out of nowhere’. Really the issue here is not what is in the statistical analyses section but the foregoing information in the paper. There is a lack of build-up and explanation in the intro to prepare readers for these analyses and they are not clear in the aims/objectives of the study.

*Authors’ response: We agree with the reviewer that these analyses should have been more clearly introduced in the Introduction section of the paper.*

*Changes to the manuscript: please see p. 4, lines 72-74 and p. 5, 101-105 of the track changes version of the manuscript.*

11. Halfway through a paragraph in the statistical analyses section, a factor analysis is mentioned with minimal information. If the purpose of the factor analysis is to develop the main tool which is used in the paper (which it is) I would expect much, much more information about this – the type of analysis done, the scree plot, the factor loadings etc. As it is, we don’t know from the manuscript even how many items the authors started with and the approach they used to reducing the number of items they had. This is very concerning. I would expect to see all the original items in a table with their factor loadings and then the approach used for reducing these. I’d also expect to see some kind of justification for the sample size.

*Authors’ response: We apologize for any confusion. In contrary to the reviewer’s comment, the factor analysis was not performed “to develop” the main tool, but rather to evaluate the psychometric properties of the tool after the first round of data collection was performed. The data collection tool was developed rapidly based on literature review and expert opinion (as explained above and in p. 6-7, lines 128-139 of the Methods section). As mentioned in the paper, no formal sample size was performed before the start of this study (see p. 9, lines 189-190).*

*Changes to the manuscript: We have added the following to the paragraph to provide further explanation for the use of principal component factor analysis, which was used to develop variables measuring beliefs about PPE effectiveness, PPE availability, skills, and institutional trust in the multivariable models (see p. 8-9, lines 176-188).*

From the Likert-scale questions, we conducted principal component factor analysis to examine correlation matrices and construct indices from the first principal component for the following constructs: beliefs about effectiveness of PPE, availability of PPE at the respondent’s institution, skills, and institutional trust test. The first principal component for each of these constructs was then included as an explanatory variable in the multivariable analysis. This allowed us to test, for example, for the independent effect of institutional trust, on the dependent variable, HCWs’ perceived sense of control over getting infected with COVID-19, while control for other regression model covariates. For each of the constructed indices, Cronbach’s alpha scores for the Likert-scale components exceeded 0.75.

12. Information about how much data was missing and how this was managed needs to be provided in the statistical analyses section

*Authors’ response: we thank the reviewer for this important comment. As mentioned in the Methods section, surveys with completion of only the first part of the survey containing demographic and basic information about IPC training (corresponding to survey completion <58%) were excluded from data analyses. Due to mid- or end-survey dropout, the denominator per statement is different. Therefore, the total number of respondents per statement is provided in Fig 1, and if otherwise, in the manuscript body. For example in case of perceptions about emotional wellbeing, the sentence “There were 2180 (95%) HCWs who completed all questions about emotional wellbeing” is provided. In case of other denominators (for example when analyzing subgroups), this is also mentioned specifically in the manuscript text. We agree with the reviewer that this information should also be available in the Methods section.*

*Changes to the manuscript: please see p. 9, lines 192-194 of the track changes version of the manuscript).*
