# Supplementary material for: The experience of European hospital-based health care workers on following infection prevention and control procedures and their wellbeing during the first wave of the COVID-19 pandemic
Source: PLoS One. 2022 Feb 7;17(2):e0245182. doi: 10.1371/journal.pone.0245182 (PMC8820620; doi:10.1371/journal.pone.0245182)
Supplement: Attachment — Submitted filename: 20220110 Response to reviewers.docx [file pone.0245182.s008.docx]

**To**:	Dr. Amitava Mukherjee

Academic Editor of *PLOS ONE*

**Date**:	January 10, 2022

Utrecht, The Netherlands

Dear Dr. Amitava Mukherjee,

We sincerely thank the reviewer for their valuable time to evaluate our manuscript. The paper has been modified in response to the comments and suggestions. All changes are highlighted in the track changes version of the manuscript. In the following we address all reviewer’s comments point by point. This response letter has also been uploaded as a separate document.

Many thanks in advance. On behalf of all authors,

Yours sincerely,

Denise van Hout, MD, PhD

[denise.van.hout@rivm.nl](mailto:denise.van.hout@rivm.nl)

Reviewer #2: This manuscript assesses the perception of hospital HCWs on the adequacy of local IPC measures and concludes that institutions should adopt a multifaceted approach in IPC preparedness and training in order to best support hospital HCWs at work during an infectious diseases pandemic. The authors discuss factors that contribute to staff wellbeing and the perceived efficacy of PPE and the results are clearly presented.

*Author’s response: We would sincerely like to thank the reviewer for their valuable time and we sincerely appreciate the careful reading of our manuscript. In the following we will address all comments point by point.*

Minor comments: While a PCA is mentioned in the methods section, there are no details on the results of the PCA and a more detailed description of this analysis should be added as a supplementary file.

*Author’s response: we thank the reviewer for this comment. The principal component factor analysis was performed to construct indices from the first principal component for the following measures: beliefs about effectiveness of PPE, availability of PPE at the respondent’s institution, and skills for preparedness for dealing with COVID-19. The index constructed from the first principal component for each of these measures was then included as an explanatory variable in the multivariable analysis. We agree with the reviewer that details on data-analysis and results of the PCA were missing in the manuscript.*

*Changes to the manuscript: details on the PCA were clarified in the main paper (please see p. 8, lines 168-174), and a detailed description of the PCA was added as a new supplementary file – please see “S2 File”.*

The background section of the abstract would be strengthened if the rational behind the study was discussed in more detail.

*Changes to the manuscript: To clarify the rationale of the study in the abstract, we have updated the text – please see p. 2, lines 29-31 of the revised manuscript.*
